# Preclinical characterization of WB737, a potent and selective STAT3 inhibitor, in natural killer/T‐cell lymphoma

**DOI:** 10.1002/mco2.284

**Published:** 2023-06-16

**Authors:** Yali Wang, Wenbo Zhou, Jianfeng Chen, Jinghong Chen, Peng Deng, Huang Chen, Yichen Sun, Zhaoliang Yu, Diwen Pang, Lizhen Liu, Peili Wang, Jing Han Hong, Bin Tean Teh, Huiqiang Huang, Wenyu Li, Zhengfang Yi, Soon Thye Lim, Yihua Chen, Choon Kiat Ong, Mingyao Liu, Jing Tan

**Affiliations:** ^1^ State Key Laboratory of Oncology in South China Collaborative Innovation Center of Cancer Medicine Sun Yat‐Sen University Cancer Center Guangzhou China; ^2^ Shanghai Key Laboratory of Regulatory Biology Institute of Biomedical Sciences and School of Life Sciences East China Normal University Shanghai China; ^3^ Shanghai Yuyao Biotech Co., Ltd. Shanghai China; ^4^ Department of Medical Oncology The Affiliated Cancer Hospital of Zhengzhou University & Henan Cancer Hospital Zhengzhou China; ^5^ Department of Laboratory Medicine Guangzhou First People's Hospital School of Medicine South China University of Technology Guangzhou China; ^6^ Department of Colorectal Surgery The Sixth Affiliated Hospital Sun Yat‐Sen University Guangzhou China; ^7^ Guangdong Provincial People's Hospital Guangdong Academy of Medical Sciences School of Medicine South China University of Technology, Guangzhou China; ^8^ Cancer and Stem Cell Biology Program Duke‐NUS Medical School Singapore; ^9^ Laboratory of Cancer Epigenome Division of Medical Sciences National Cancer Centre Singapore Singapore; ^10^ Director's Office, National Cancer Centre Singapore Singapore; ^11^ Division of Cellular and Molecular Research National Cancer Centre Singapore Singapore

**Keywords:** activating mutations, inhibitor, MYC, NKTL, oxidative phosphorylation, STAT3

## Abstract

Natural killer/T‐cell lymphoma (NKTL) is an uncommon malignancy with poor prognosis and limited therapeutic options. Activating mutations of signal transducer and activator of transcription 3 (STAT3) are frequently found in patients with NKTL, suggesting that targeted inhibition of STAT3 is a potential therapeutic option for this disease. Here, we have developed a small molecule drug WB737 as a novel and potent STAT3 inhibitor that directly binds to the STAT3‐Src homology 2 domain with high affinity. In addition, the binding affinity of WB737 to STAT3 is 250‐fold higher than STAT1 and STAT2. Interestingly, WB737 is more selective for NKTL with *STAT3*‐activating mutations in terms of growth inhibition and apoptotic induction when compared with Stattic. Mechanistically, WB737 inhibits both canonical and noncanonical STAT3 signaling via suppression of STAT3 phosphorylation at Tyr705 and Ser727, respectively, thereby inhibiting the expression of c‐Myc and mitochondria‐related genes. Moreover, WB737 inhibited STAT3 more potently than Stattic, resulting in a significant antitumor effect with undetectable toxicity, followed by almost complete tumor regression in an NKTL xenograft model harboring a *STAT3*‐activating mutation. Taken together, these findings provide preclinical proof‐of‐concept for WB737 as a novel therapeutic strategy for the treatment of NKTL patients with *STAT3*‐activating mutations.

## Graphical abstract

WB737 is a novel, potent, and selective STAT3 inhibitor. WB737 suppresses the level of pTyr705‐STAT3, thereby inhibiting its transcriptional activity. In addition, WB737 also inhibits the levels of pSer727‐STAT3 and genes encoding core components of the electron transport chain, thereby impairing respiratory complex I activity and resulting in a strong inhibition of oxidative phosphorylation.

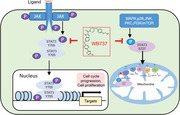

## 1 INTRODUCTION

Natural killer/T‐cell lymphoma (NKTL) is a rare and aggressive hematological malignancy with geographical prevalence in East Asia.^1^ The disease is more prevalent in males than females and is strongly correlated to Epstein‒Barr virus infection.^2^, ^3^ Approximately 80% of NKTL occurs in the nose and upper aerodigestive tract (nasal‐type NKTL), while 20% of NKTL occurs in non‐nasal areas (extranasal NKTL).^4^ Despite the effectiveness of combining chemotherapy and radiotherapy for early‐stage NKTL, the survival outcomes remain dismal, with 5‐year overall survival (OS) rates of 54% and 34% for nasal and extranasal NKTL, respectively.^5^, ^6^ Patients with recurrent or advanced stage disease are usually resistant to treatments, and their 5‐year OS is less than 20%.^7^ Therefore, there is an unmet clinical need for an effective targeted therapy, especially for patients with relapsed or refractory NKTL.

Signal transducer and activator of transcription 3 (STAT3) is an important transcription factor involved in multiple biological processes, including cell growth, survival, and cell cycle progression. Constitutive activation of STAT3 is frequently found in various malignancies, including NKTL.^8^, ^9^ STAT3 has been implicated as an oncogene by promoting cell proliferation, metastasis, angiogenesis, and immune evasion.^10^ STAT3 is activated by phosphorylation at tyrosine 705 (pTyr705), which is catalyzed by Janus kinases (JAK) and other tyrosine kinases. Activated STAT3 forms parallel dimers through interaction with its Src homology 2 (SH2) domain and subsequently translocates to the nucleus, binding to DNA to regulate gene transcription,^11^ while the antiparallel dimers of unphosphorylated STAT3 can also translocate to the nucleus and bind to DNA.^12^ In addition to this canonical pathway, STAT3 can act as an oncogenic driver through noncanonical STAT3 signaling. STAT3 is activated via the phosphorylation of STAT3 at Serine 727 (pSer727‐STAT3) and is subsequently localized into mitochondria and promotes mitochondrial respiration, which in turn drives tumor growth.^13^, ^14^ Recent studies have identified *STAT3* as one of the most frequently mutated genes in NKTL, with a mutation rate from 10% to 22%.^15^, ^16^, ^17^ Moreover, hotspot mutations of *STAT3* are frequently located within the SH2 domain, which induces hyperactivation of STAT3 signaling via constitutive phosphorylation of STAT3 at Y705 (pTyr705‐STAT3) and contributes to NKTL pathogenesis,^18^, ^19^, ^20^, ^21^, ^22^ suggesting that STAT3 is a promising target for treating patients with NKTL. However, inhibition of STAT3 by small molecules is challenging mainly due to the lack of enzymatic activity in STAT3.^23^

There are three potential strategies to target the STAT3 signaling pathway: inhibiting upstream receptors and kinases, impeding STAT3 activation or function, and sequestering STAT3 away from DNA‐binding sites.^24^Ruxolitinib, a dual JAK1/JAK2 inhibitor, significantly reduces spleen size and symptoms in myelofibrosis, but its potential adverse effects, such as increased risks of neutropenia, infection, and anemia, limit its clinical application.^25^, ^26^, ^27^ Although STAT3‐SH2 domain inhibitors, including Stattic, OPB‐31121, and OPB‐51602, have shown promising efficacy in patients with advanced solid malignancies, they are limited by suboptimal potency, poor stability, and life‐threatening toxicities.^28^, ^29^, ^30^ In addition, therapeutic targeting of STAT3 with antisense oligonucleotides, AZD9150, or decoy oligonucleotides remains technically challenging due to its rapid degradation.^30^, ^31^ Thus, there is an unmet clinical need to develop novel and potent STAT3 inhibitors.

Here, we identified WB737 as a small molecule STAT3 inhibitor using molecular docking, microscale thermophoresis (MST), and STAT3‐response luciferase reporter assays. We demonstrated that constitutively activating *STAT3* mutations confer sensitivity to WB737 in NKTL. Mechanistically, we found that WB737 selectively inhibits *STAT3* ^Mut^ NKTL growth by suppressing both the JAK–STAT–MYC signaling pathway and the oxidative phosphorylation pathway. More importantly, we showed that WB737 treatment resulted in a strong antitumor effect followed by complete tumor regression in *STAT3* ^Mut^ NKTL in vivo. In summary, our data suggest that WB737, a novel STAT3 inhibitor, holds potential therapeutic value for a selected subset of NKTL patients with *STAT3*‐activating mutations.

## 2 RESULTS

### 2.1 Characterization of WB737 as a potent and novel STAT3 inhibitor

**FIGURE 1:**
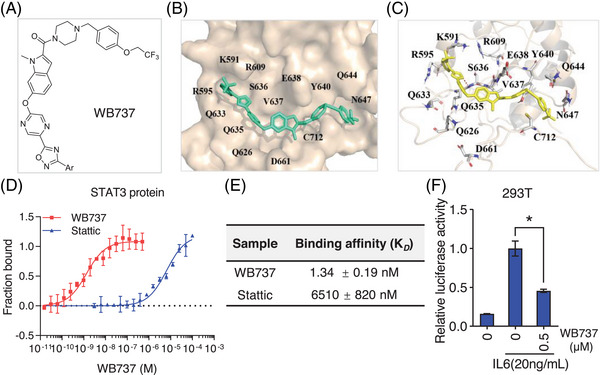
In vitro characterization identified WB737 as a potent signal transducer and activator of transcription 3 (STAT3) inhibitor. (A) Chemical structure of WB737. (B and C) A molecular docking model revealed that WB737 binds to the Src homology 2 (SH2) domain of STAT3. (D) Microscale thermophoresis (MST) assays. WB737 and Stattic were serially diluted and mixed with equal volumes of labeled His‐STAT3^127‐722^ protein. The MST signal was measured, and the data were analyzed using MO affinity analysis software. (E) The table displays the binding affinity between the STAT3 protein and STAT3 inhibitors (WB737 and Stattic). (F) Transient transfection of 293T cells with a STAT3‐dependent luciferase reporter plasmid was performed for 24 h, and the cells were treated with WB737 for 24 h with or without interleukin‐6 (IL‐6; 20 ng/mL). Renilla luciferase activities were used as an internal reference (*n* = 2 per group). The values are expressed as the mean ± standard deviation (SD), and the bars indicate statistically significant differences (*t*‐test). ^*^ *p* < 0.05.

In our previous studies,^32^, ^33^ we have used three functional assays to screen inhibitors that abolished STAT3 pTyr705 and pSer727 concurrently, including the MST assay (based on affinity for STAT3 protein), the STAT3‐luciferase reporter assay (based on pTyr705 nuclear transcriptional function), and the mitochondrial respiration assay (based on pSer727 mitochondrial oxidative phosphorylation [OXPHOS] function). Interestingly, our preliminary screening results showed that a small molecule, WB737, exhibited the best inhibitory activity among the investigated compounds. Therefore, WB737 was discovered as a novel small molecule STAT3 inhibitor (Figure 1A). The high‐resolution mass spectrum (HRMS) of WB737 was collected on a Bruker MicroTOF‐Q III LC–MS instrument operating in electrospray ionization. The *m*/*z* values for WB737 in HRMS were 760.2077 and 760.2085 (Figure S1A). Computational docking assays showed that STAT3 (PDB ID: 1BG1) complexed with WB737 (Figure 1B) at multiple amino acid residues in STAT3, such as Lys591 and Arg 609 (Figure 1C), which are critical for the interaction of phosphotyrosine 705 with the STAT3‐SH2 domain.^34^ An MST assay was performed to quantify the interaction between WB737 and the STAT3‐SH2 domain. The results indicated that WB737 interacted with STAT3^127‐722^ in a dose‐dependent manner with a *K*_D_ of 1.34 ± 0.19 nM (Figure 1D,E). In addition, we performed the same MST assay using Stattic, a previously described SH2 inhibitor.^35^ The data showed that Stattic had a *K*_D_ of 6.51 ± 0.82 μM (Figure 1D,E), suggesting that it binds less effectively to the STAT3‐SH2 domain than WB737, blocking pTyr705. Moreover, to determine whether WB737 selectively bound to STAT3 over STAT family proteins, we purified STAT family proteins (STAT1 and STAT2) and performed direct binding assays between WB737 and STAT1 or STAT2 in MST assays. Our data showed that both STAT1 and STAT2 proteins had *K*_D_ values greater than 500 nM (Figure S1B,C), indicating that WB737 is more selective for STAT3 than for STAT1 and STAT2. To further test the bioactivity of WB737, we carried out a luciferase reporter assay in 293T cells in the presence of interleukin‐6 (IL‐6). Our data showed that IL‐6 stimulation significantly induced STAT3‐dependent luciferase reporter activity, which was significantly suppressed after WB737 treatment (Figure 1F). Collectively, these data demonstrated that WB737 bound to the STAT3‐SH2 domain with high affinity and is a potent inhibitor of STAT3 transcriptional activity.

### 2.2 Constitutive *STAT3*‐activating mutations confer sensitivity of NKTL to WB737

**FIGURE 2:**
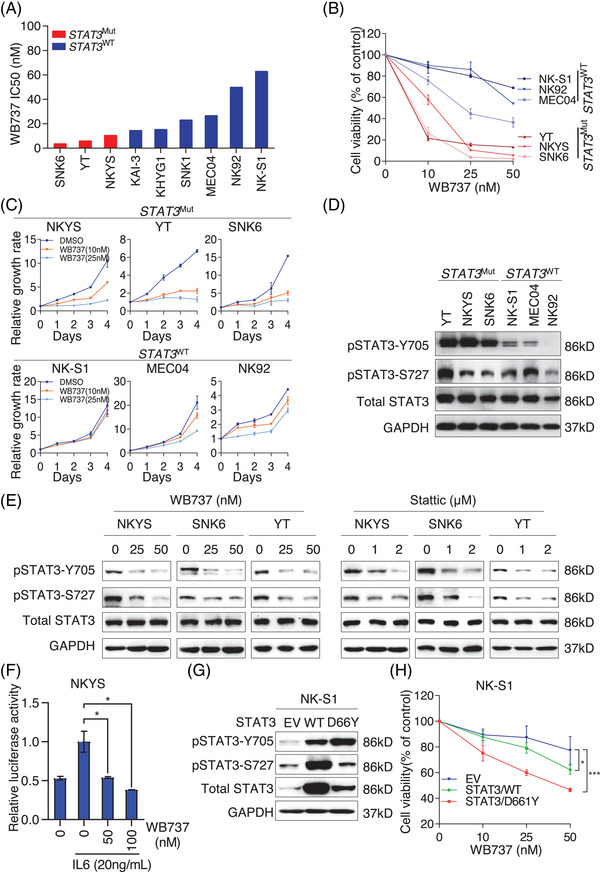
Constitutive signal transducer and activator of transcription 3 (STAT3)‐activating mutations confer sensitivity of natural killer/T‐cell lymphoma (NKTL) to WB737. (A) Half maximal inhibitory concentrations (IC_50_) of WB737 and *STAT3* mutation status in nine NKTL cell lines (red, *STAT3*‐activating mutations; blue, wild‐type STAT3). (B) Cell viability of three pairs of NKTL cell lines (STAT3^WT^, NK‐S1, NK92, MEC04; STAT3^Mut^, YT, NKYS, SNK6) treated with the indicated concentrations of WB737 for 96 h. Cell viability was normalized to the control, dimethyl sulfoxide (DMSO) and presented as the mean ± standard deviation (SD). (C) Growth curves of NKTL cell lines in the presence of WB737. Upper panel: *STAT3* ^Mut^ cells; lower panel: *STAT3* ^WT^ cells. (D) Immunoblot analysis of the indicated proteins in *STAT3* ^Mut^ and *STAT3* ^WT^ cells. (E) Western blot analysis of pTyr705‐STAT3, pSer727‐STAT3, and total STAT3 expression in *STAT3* ^Mut^ and *STAT3* ^WT^ cells treated with the indicated concentrations of WB737 or Stattic. (F) A STAT3‐specific luciferase reporter plasmid was transiently transfected into NKYS cells. After 24 h of transfection, WB737 was added at the indicated concentrations with or without interleukin‐6 (IL‐6; 20 ng/mL). Renilla luciferase activities were used as an internal reference (*n* = 2 per group). Data are expressed as the mean ± SD, and the bars indicate statistically significant differences (^*^ *p* < 0.05, *t*‐test). (G) Immunoblot assessment of the levels of pTyr705‐STAT3, pSer727‐STAT3, and total STAT3 in NK‐S1 isogenic cells with empty vector (EV), wild‐type STAT3 (STAT3/WT), and mutant STAT3 (STAT3/D661Y). (H) Dose‐dependent response of NK‐S1 isogenic cells to WB737 treatment. Cell viability was normalized to the control (DMSO) and is presented as the mean ± SD. ^*^ *p* < 0.05, ^***^ *p* < 0.001.

Frequent somatic mutations of STAT3 have been demonstrated in NKTL, and silencing of STAT3 signaling was shown to inhibit cell proliferation.^18^, ^24^ To determine the in vitro efficacy of WB737 in NKTL, we evaluated the half maximal inhibitory concentrations (IC_50_) of WB737 in a panel of nine NKTL cell lines. The data showed that WB737 displayed cytotoxicity in the nanomolar range in all NKTL cells (Figure 2A), indicating that WB737 induced strong growth inhibition in NKTL. Intriguingly, NKTL cell lines harboring *STAT3*‐activating mutations (*STAT3* ^Mut^), such as SNK6, YT, and NKYS, were more sensitive to WB737 than those with wild‐type *STAT3* (*STAT3* ^WT^), as demonstrated by the lower IC_50_ (Figure 2A). Consistently, WB737 effectively suppressed cell viability in the three *STAT3* ^Mut^ NKTL cell lines (SNK6, YT, and NKYS) in a dose‐dependent manner, whereas limited effects were detected in those with *STAT3* ^WT^ (NK‐S1, MEC04, and NK92) (Figure 2B). In addition, WB737 significantly inhibited cell growth in three *STAT3* ^Mut^ cell lines compared to the three *STAT3* ^WT^ cell lines (Figure 2C). To further investigate whether STAT3 activity is correlated with sensitivity to WB737 in NKTL, we profiled the endogenous activity of STAT3 in three *STAT3* ^WT^ cell lines and three *STAT3* ^Mut^ cell lines. Interestingly, our data showed that constitutive pTyr705‐STAT3 was observed only in *STAT3* ^Mut^ cells but not in *STAT3* ^WT^ cells (Figure 2D). Moreover, no significant correlation between the level of pSer727‐STAT3 and STAT3 mutation status was observed (Figure 2D), suggesting that *STAT3* ^Mut^ cells may be more dependent on pTyr705‐STAT3 for survival than *STAT3* ^WT^ cells. To explore whether WB737 could inhibit STAT3 activity, we treated three *STAT3* ^Mut^ cell lines with the indicated concentrations of WB737 or Stattic. Immunoblotting data showed that WB737 inhibited both pTyr705‐STAT3 and pSer727‐STAT3 levels in a dose‐dependent fashion, whereas Stattic had a similar inhibitory effect at micromolar concentrations (Figure 2E). We next performed a dual luciferase assay in NKYS cells as previously described to examine the effect of WB737 on STAT3 transcriptional activity. Our data showed that WB737 significantly suppressed the STAT3‐response luciferase activities in a dose‐dependent fashion (Figure 2F). To further determine whether *STAT3*‐activating mutations are correlated with the sensitivity of NKTL to WB737, we ectopically expressed STAT3 wild‐type or mutant (D661Y) plasmids in NK‐S1 cells and treated these cells with the indicated concentrations of WB737. Consistent with our previous studies,^36^ compared to cells carrying empty vector (EV), cells with wild‐type STAT3 (STAT3/WT) displayed an increase in pTyr705‐STAT3, while a more significant increase was observed in cells harboring mutant STAT3 (STAT3/D661Y) (Figure 2G). Cells carrying STAT3/D661Y were more sensitive to WB737 than cells harboring EV or STAT3/WT, while the sensitivity of WB737 was correlated with the level of pTyr705‐STAT3 (Figure 2H), suggesting that constitutively activating mutations of STAT3 may confer sensitivity of NKTL to WB737. Consequently, these results prove that WB737 more effectively inhibits the growth of *STAT3* ^Mut^ NKTL cells than *STAT3* ^WT^ cells, probably by inhibiting the levels of pTyr705‐STAT3 and pSer727‐STAT3, suggesting that a *STAT3*‐activating mutation may represent a predictive biomarker of response to WB737 in NKTL.

### 2.3 WB737 selectively inhibits the growth of *STAT3* ^Mut^ NKTL cells by inducing apoptosis and blocking colony formation

**FIGURE 3:**
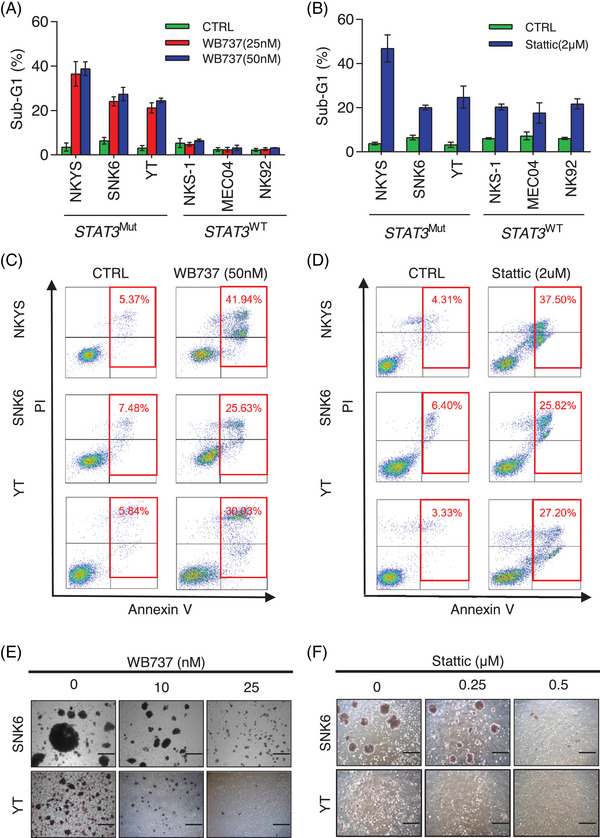
WB737 selectively inhibits the growth of STAT3Mut natural killer/T‐cell lymphoma (NKTL) cells by inducing apoptosis and blocking colony formation. (A and B) Sub‐G1 population analysis in *STAT3* ^Mut^ and *STAT3* ^WT^ cells treated with the indicated concentrations of (A) WB737 or (B) Stattic for 72 h. The results are expressed as the mean ± standard deviation (SD) from three independent experiments. (C and D) Annexin V staining of three *STAT3* ^Mut^ cell lines treated with (C) WB737 or (D) Stattic for 72 h. (E and F) Colony formation assay in *STAT3* ^Mut^ cells (SNK6 and YT) treated with the indicated concentrations of (E) WB737 or (F) Stattic for 10 days. DMSO‐treated cells served as controls in all experiments. Scale bar = 200 μm. STAT3, signal transducer and activator of transcription 3.

Next, we performed several cellular assays in vitro to assess the efficacy of WB737 in *STAT3* ^WT^ and *STAT3* ^Mut^ cells. Treatment of cells with WB737 at nanomolar concentrations selectively induced cell death in *STAT3* ^Mut^ cell lines but not in *STAT3* ^WT^ cell lines (Figure 3A). However, at an effective concentration of 2 μM, Stattic induced cell death equally in both *STAT3* ^Mut^ and *STAT3* ^WT^ cell lines (Figure 3B), suggesting that WB737 is more selective for *STAT3* ^Mut^ cells than Stattic. Annexin V staining analysis revealed that both WB737‐ and Stattic‐induced apoptosis are the main mechanisms of cell death in these *STAT3* ^Mut^ cells (Figure 3C,D). Moreover, WB737 inhibited the clonogenic potential of *STAT3* ^Mut^ cells (SNK6 and YT) at low nanomolar concentrations in a dose‐dependent manner (Figure 3E), while similar results were only obtained when Stattic was used at micromolar concentrations 10–25‐fold higher than WB737 (Figure 3F). These data indicate that WB737 selectively inhibits tumorigenesis of *STAT3* ^Mut^ NKTL cells by inducing apoptosis and blocking colony formation.

### 2.4 WB737 selectively targets canonical and noncanonical STAT3 signaling in *STAT3* ^Mut^ NKTL cells

**FIGURE 4:**
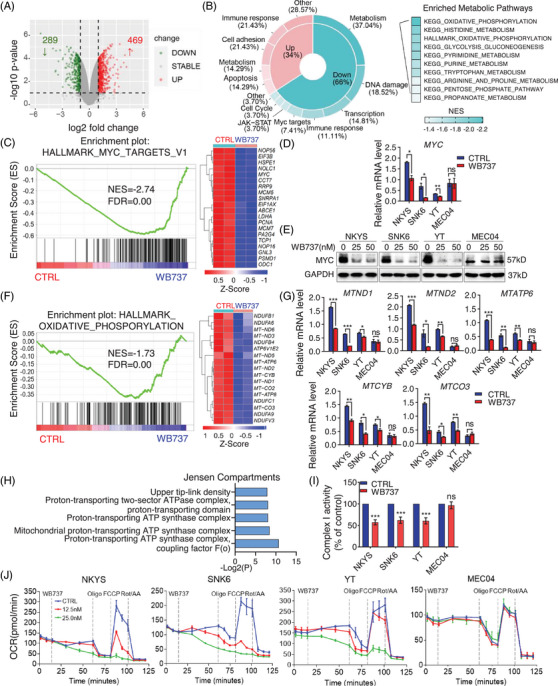
Targeting canonical and noncanonical signal transducer and activator of transcription 3 (STAT3) signaling pathways by WB737 in STAT3Mut natural killer/T‐cell lymphoma (NKTL). (A) Volcano plot of differentially expressed genes in NKYS cells treated with WB737 (50 nM) compared to control cells. (B) Gene set enrichment analysis (GSEA) using the Kyoto Encyclopedia of Genes and Genomes (KEGG) database revealed the significantly up‐ and downregulated pathways in NKYS cells after WB737 treatment. (C) GSEA using the Hallmarks pathway database showed that the Myc‐Targets‐V1 pathway was significantly enriched in the downregulated genes (left panel). Heatmap reveals the top 20 downregulated genes of Myc‐Targets‐V1 pathways in NKYS after WB737 treatment (right panel). (D) RT‒qPCR analysis of *MYC* in *STAT3* ^Mut^ (NKYS, SNK6, and YT) and *STAT3* ^WT^ (MEC04) cells treated with WB737. (E) Immunoblotting analysis of the indicated proteins in *STAT3* ^Mut^ (NKYS, SNK6, and YT) and *STAT3* ^WT^ (MEC04) cells treated with WB737 for 24 h. (F) GSEA using the Hallmarks pathway database revealed that the oxidative phosphorylation (OXPHOS) pathway was significantly enriched in the downregulated genes (left panel). The heatmap indicates the top 20 downregulated genes of the OXPHOS pathway in NKYS cells treated with WB737 (right panel). (G) RT‒qPCR analysis of *MTND1*, *MTND2*, *MTCYB*, *MTCO3*, and *MTATP6* in *STAT3* ^Mut^ (NKYS, SNK6, and YT) and *STAT3* ^WT^ (MEC04) cells treated with WB737. (H) Top five significantly enriched compartments in genes related to the OXPHOS pathway downregulated by WB737 in NKYS. (I) Complex I activity of *STAT3* ^Mut^ (NKYS, SNK6, and YT) and *STAT3* ^WT^ (MEC04) cells in the absence or presence of WB737. Complex I activity is normalized to the control (DMSO) and presented as the mean ± standard deviation (SD). (J) Oxygen consumption rate (OCR) in *STAT3* ^Mut^ (NKYS, SNK6, and YT) and *STAT3* ^WT^ (MEC04) cells treated with the indicated concentrations of WB737. Data are representative of three independent experiments with similar results. Error bars represent the mean ± SD. ^*^ *p* < 0.05, ^**^ *p* < 0.01, ^***^ *p* < 0.001, two‐tailed Student's *t‐*test (D, G, and I). NES, normalized enrichment score; FDR, false discovery rate (B, C, and F). *Z*‐score denotes the deviation from the mean by standard deviation units. Red color indicates upregulated expression, whereas blue color indicates downregulated expression (C and F).

To investigate the precise mechanism underlying sensitivity to WB737 in *STAT3* ^Mut^ NKTL cells, we performed RNA sequencing in NKYS cells treated with WB737. Transcriptomic profiling analysis identified that WB737 upregulated 469 genes and downregulated 289 genes in NKYS cells (Figure 4A). Gene set enrichment analysis (GSEA) using the Kyoto Encyclopedia of Genes and Genomes (KEGG) database revealed that immune response pathways were enriched in the upregulated genes (Figure 4B), including NK cell‐mediated cytotoxicity pathway (Figure S2A), which is consistent with previous studies showing that inhibition of oncogenic STAT3 signaling enhances the NK cell‐mediated cytotoxicity.^37^, ^38^ More importantly, the top enriched gene sets of the downregulated genes included the OXPHOS, JAK–STAT, and Myc target pathways (Figure 4B). Consistently, GSEA using the Hallmarks pathway database also showed that Myc‐Targets‐V1 and OXPHOS pathways were significantly enriched in the downregulated genes (Figure 4C,F). Previous studies demonstrated that c‐Myc is a critical target gene of the canonical JAK–STAT3 pathway,^39^, ^40^ which was further confirmed in our study (Figure 4C). WB737 significantly inhibited the expression of pTyr705‐STAT3 in *STAT3* ^Mut^ cells (Figure 2E), thereby inhibiting the expression level of c‐Myc (Figure 4D,E). However, WB737 had little effect on the expression of c‐Myc in MEC04 (*STAT3* ^WT^) cells, as measured by RT‒qPCR and Western blot analysis (Figure 4D,E). Intriguingly, more than 37% of the downregulated genes were enriched in metabolic signaling pathways (Figure 4B,F), suggesting that WB373 may also inhibit noncanonical STAT3 signaling. Previous studies have reported that pSer727‐STAT3 localized in mitochondria enhances OXPHOS signaling by increasing the transcription of mitochondrial genes or facilitating the activity of complex I in the electron transport chain (ETC).^41^, ^42^ To confirm whether WB737 could suppress both the transcription of mitochondrial genes and the activity of complex I, thereby inhibiting OXPHOS in *STAT3* ^Mut^ cells, we first determined the effect of WB737 on the transcription of mitochondrial genes. Our data showed that WB737 significantly suppressed the expression of mitochondrial genes in *STAT3* ^Mut^ cells, including *MTND1*, *MTND2*, *MTCYB*, *MTCO3*, and *MTATP6*, which encode components of the ETC. However, WB737 had little or no effect in MEC04 cells (Figure 4G), suggesting that WB737 selectively inhibited OXPHOS in *STAT3* ^Mut^ but not in STAT3^WT^ cells. In addition, the involvement of genes downregulated by WB737 in OXPHOS was confirmed using the Jensen Compartments library (Enrichr databases),^43^ indicating that four of the top five significantly enriched compartments were associated with the mitochondrial proton‐transporting adenosine triphosphate (ATP) synthase complex (Figure 4H). To further determine whether WB737 could inhibit the activity of complex I of the ETC, we performed a complex I enzyme activity assays in *STAT3* ^WT^ (MEC04) and *STAT3* ^Mut^ (NKYS, SNK6, and YT) cell lines. The data showed that WB737 significantly reduced the activity of complex I in *STAT3* ^Mut^ cell lines, while little change was observed in MEC04 cells (Figure 4I). Moreover, we performed Seahorse experiments to assess whether the oxygen consumption rate (OCR) could be suppressed by WB737 in these cells. Consistently, our data revealed that WB737 dose‐dependently inhibited OCR in *STAT3* ^Mut^ cells, but not in MEC04 cells (Figure 4J). Together, these findings demonstrated that WB737 selectively inhibited both canonical and noncanonical STAT3 signaling through suppression of pTyr705‐STAT3 and pSer727‐STAT3 in *STAT3* ^Mut^ cells, suggesting that WB737 may represent a potential therapeutic strategy in NKTL patients with *STAT3*‐activating mutations.

### 2.5 Concurrent inhibition of STAT3 phosphorylation at Tyr705 and Ser727 by WB737 potently induced tumor regression in vivo

**FIGURE 5:**
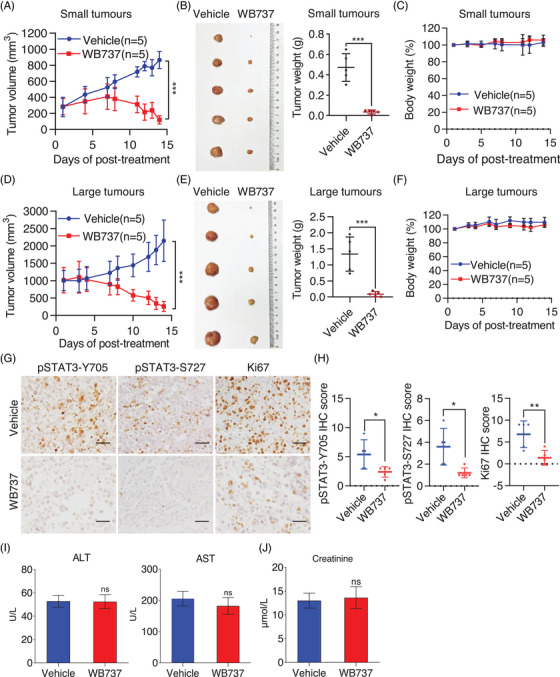
In vivo administration of WB737 induces almost complete tumor regression in STAT3Mut natural killer/T‐cell lymphoma (NKTL). (A) Growth curve of YT xenografts (average tumor volume [TV] 200−300 mm^3^) in nude mice treated with vehicle or WB737 at 5 mg/kg for 14 days. Error bars represent the mean ± standard error of mean (SEM) (*n* = 5 per group). ^***^ *p* < 0.001 (independent *t*‐test). (B) Representative photographs of tumor size in (A) after 14 days of treatment (left panel). Graph showing the tumor weight in (A) on the 14th day post‐treatment (right panel). Data are presented as the mean ± standard deviation (SD). ^***^ *p* < 0.001. (C) Mice bearing small YT tumors were treated with vehicle or WB737 at 5 mg/kg for 14 days, and body weight was measured twice a week. Error bars represent the mean ± SEM (*n* = 5 per group). (D) Growth curve of large YT tumors (average TV 1000 mm^3^) in nude mice with different treatments. Error bars represent the mean ± SEM (*n* = 5 per group). ^***^ *p* < 0.001 (independent *t*‐test). (E) Photographs showing tumor size in (D) after treatment with WB737 for 14 days (left panel). The tumor weight in (D) on the 14th day post‐treatment (right panel). Data are presented as the mean ± SD. ^***^ *p* < 0.001. (F) Mice bearing large YT tumors were treated with vehicle or WB737 at 5 mg/kg for 14 days, and body weight was monitored twice a week. Error bars represent the mean ± SEM (*n* = 5 per group). (G) Representative immunohistochemistry (IHC) images of pTyr705‐STAT3, pSer727‐STAT3, and Ki67 in xenografts derived from YT cells. Scale bar = 25 μm. (H) IHC score of the level of pTyr705‐STAT3, pSer727‐STAT3, and Ki67 in tumor sections with different treatments. (I) Liver function tests were performed after treatment to evaluate alanine aminotransferase (ALT) and aspartate aminotransferase (AST) in different treatment groups. Data are presented as the mean ± SD. (J) Serum creatinine levels in the vehicle and WB737 groups. Data are presented as the mean ± SD. STAT3, signal transducer and activator of transcription 3.

To evaluate the clinical potential of WB737, we examined its exposure post oral (10 mg/kg, p.o.) and intravenous administration (1 mg/kg, i.v.). Our data showed that the half‐lives (*t*_1/2_) of WB737 in mice after oral and intravenous administration were 14.70 and 29.08 h, respectively. The oral bioavailability of WB737 in mice was 38.46% (Figure S3A). We also detected the stability of WB737 in both plasma and liver microsomes from mice. Our results showed that the *t*_1/2_ of WB737 was 911.03 min (Figure S3B,C). These data suggested that WB737 has good oral bioavailability and plasma stability in mice. Therefore, the p.o. route is preferred for administration of WB737. To determine the ability of WB737 to exert its anti‐STAT3 inhibitory effect in vivo, we assessed the antitumorigenic efficacy of WB737 in a YT subcutaneous xenograft model. When the average tumor volume (TV) reached 200−300 mm^3^, mice bearing tumors were divided into two groups: vehicle and WB737. WB737 was administered by oral gavage at 5 mg/kg daily for 14 days. The data showed that tumors were almost completely regressed when compared with those with vehicle (Figure 5A,B). To further assess whether WB737 could effectively inhibit the progression of large YT tumors (average TV 1000 mm^3^) in vivo, we established YT subcutaneous xenograft models with large tumors and then divided them into two groups: vehicle and WB737. Intriguingly, WB737 treatment caused almost complete regression of the large tumors (Figure 5D,E). We next examined whether WB737 inhibited YT xenografts mainly through inhibition of STAT3 signaling. Immunohistochemistry (IHC) analysis showed that WB737 inhibited both pTyr705‐STAT3 and pSer727‐STAT3, while markedly decreased Ki‐67 was observed with WB737 treatment compared to vehicle (Figure 5G,H). Importantly, no significant reduction in body weight was monitored (Figure 5C,F). Besides, we performed liver function tests to evaluate liver enzymes, such as alanine aminotransferase and aspartate aminotransferase, in the vehicle and WB737 groups. The data showed that WB737 did not induce hepatic toxicity after treatment with WB737 for 14 days (Figure 5I), and there was no significant change in serum creatinine levels after WB737 treatment when compared with the vehicle group (Figure 5J), indicating that the side effects of WB737 in vivo were tolerable. Accordingly, these data indicate that blockage of STAT3 by WB737 is an effective and safe treatment option for NKTL with *STAT3*‐activating mutations.

## 3 DISCUSSION

NKTL is a highly aggressive malignancy with dismal treatment outcomes at advanced stages, suggesting that novel and effective targeted therapies are urgently needed. Frequent activating mutations within the STAT3‐SH2 domain contribute to NKTL pathogenesis. Hence, STAT3 is considered a promising therapeutic target in NKTL. However, there is still no clinically available STAT3 inhibitor. Here, we discovered a novel STAT3 inhibitor, WB737, using a molecular docking assay and proved that WB737 could bind to multiple amino acid residues in the STAT3‐SH2 domain, including Lys591, Arg609, Ser636, Val637, and Glu638. Besides, WB737 is more selective for STAT3 than other STAT family proteins such as STAT1 and STAT2. More importantly, we found that WB737 treatment resulted in almost complete tumor regression of NKTL in vivo, suggesting that WB737 could be an effective targeted therapy for patients with relapsed/refractory NKTL. Furthermore, body weight assessment, liver function and renal function tests all indicate that WB737 is tolerated in vivo. Thus, WB737 is a potential novel clinical drug for NKTL. In view of the encouraging therapeutic effect of WB737 on YT xenografts, we will further verify its efficacy and safety in patient‐derived xenografts (PDX) models or humanized NKTL models in the future.

MST and luciferase reporter assays showed that WB737 exhibited a much higher binding affinity for STAT3 than Stattic, suggesting a higher efficacy over currently used STAT3 inhibitors for NKTL. Indeed, WB737 showed low nanomolar inhibitory potencies against NKTL in vitro, which was consistent with its high affinity against STAT3. Interestingly, we found that WB737 selectively targets STAT3 with activating mutations in NKTL cells, where STAT3 is hyperactive due to aberrant phosphorylation at Tyr705. Therefore, NKTL cells with activating mutations of STAT3, such as D661Y, are likely to be more sensitive to WB737.

**FIGURE 6:**
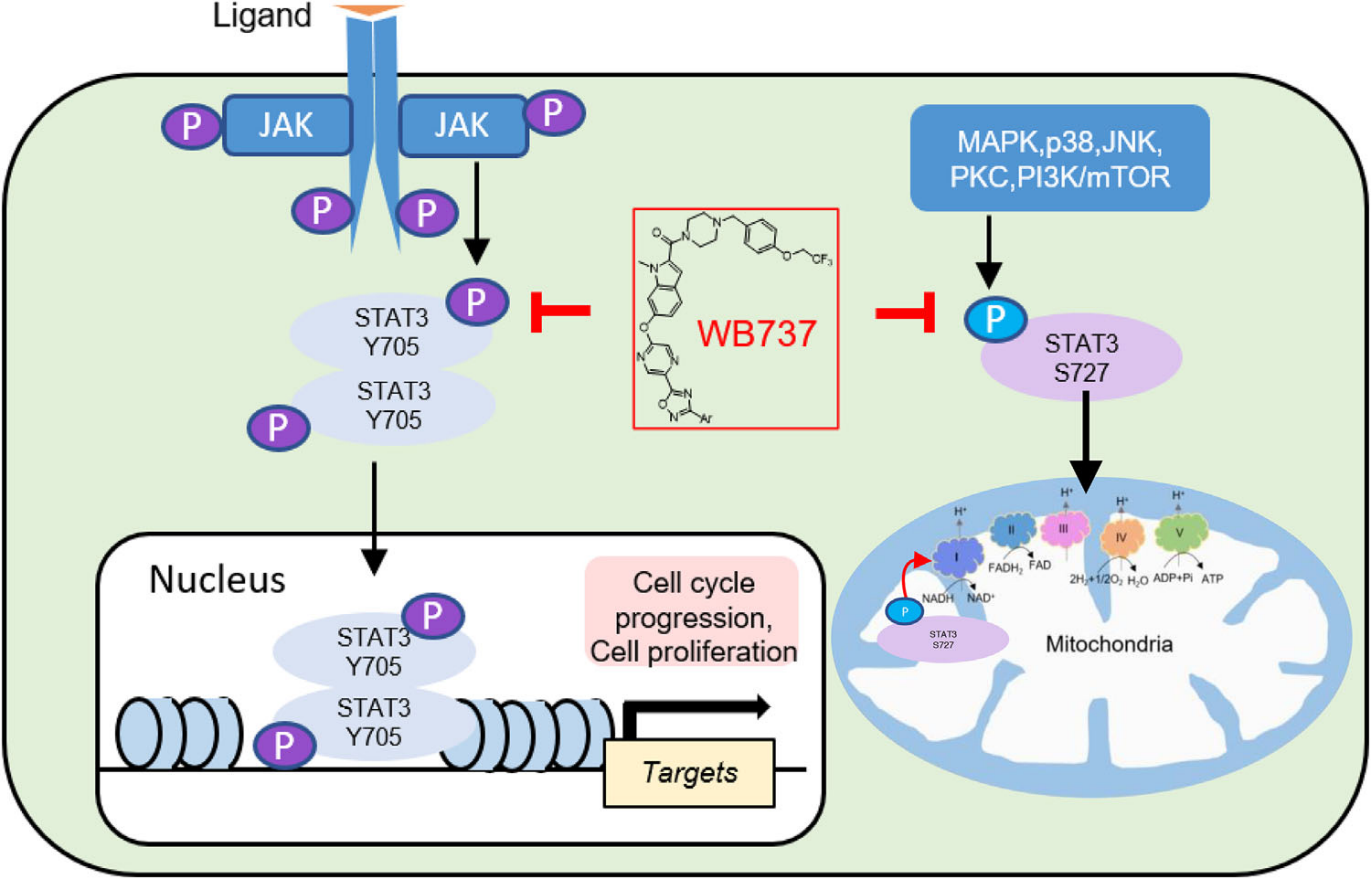
Schematic illustration of the inhibition of the canonical and noncanonical signal transducer and activator of transcription 3 (STAT3) signaling pathways by WB737. WB737 inhibited the level of pTyr705‐STAT3, thereby inhibiting the transcriptional activity of STAT3, which in turn reduced the expression of STAT3 target genes, such as MYC. In addition, targeted inhibition of pSer727‐STAT3 by WB737 not only suppressed respiratory complex I activity but also inhibited the transcription of mitochondrial genes encoding components of the electron transport chain (ETC), such as *MTND1*, *MTND2*, *MTCYB*, *MTCO3*, and *MTATP6*, resulting in significant inhibition of oxidative phosphorylation (OXPHOS).

STAT3 is involved in canonical and noncanonical STAT3 signaling pathways via phosphorylation of STAT3 at Tyr705 and Ser727, respectively. OPB‐51602 was developed as a novel STAT3 inhibitor to suppress both pTyr705‐STAT3 and pSer727‐STAT3.^44^, ^45^ OPB‐51602 has been evaluated in a clinical phase Ι trial of refractory solid and hematopoietic tumors. However, its clinical application is limited by its poor therapeutic response and tolerability.^29^, ^46^ Similarly, we found that WB737 retards both canonical and noncanonical STAT3 signaling pathways, specifically in *STAT3* ^Mut^ NKTL cells. WB737 inhibited the level of pTyr705‐STAT3, thereby suppressing the transcriptional activity of STAT3, which in turn inhibited the expression of STAT3 target genes, such as MYC. Besides, targeted inhibition of pSer727‐STAT3 by WB737 not only suppressed respiratory complex I activity but also inhibited the transcription of mitochondrial genes encoding components of the ETC, such as *MTND1*, *MTND2*, *MTCYB*, *MTCO3*, and *MTATP6*, resulting in significant inhibition of OXPHOS (Figure 6). These findings suggested that targeting OXPHOS by WB737 could be an effective therapy for patients with NKTL, as targeting OXPHOS has emerged as a potential strategy in cancer therapy.^47^, ^48^ Thus, the dual actions of WB737 result in apoptosis and reduced tumorigenic potential of *S* *TAT3* ^Mut^ NKTL cells in both in vitro and in vivo models.

However, it remains unknown why *STAT3* ^Mut^ NKTL cells are more sensitive to WB737‐induced toxicity than *STAT3* ^WT^ cells. Deeper investigation may perhaps identify new therapeutic vulnerabilities that could be exploited to target cancers that are highly dependent on JAK/STAT–MYC signaling or OXPHOS signaling for survival. Therefore, WB737 could potentially be used as a new tool to expand our knowledge of the noncanonical roles of mitochondrial STAT3 and to understand the importance of inhibiting both functions of STAT3 for effective inhibition of tumorigenesis.

STAT3 is also critical in inhibiting antitumor immune response by suppressing the expression of tumor immune activators and promoting the production of immunosuppressive factors.^49^ Of note, many studies have shown that STAT3 hyperactivation inhibits the expression of molecules related to NK cell activation to promote immune evasion,^50^ suggesting that blockade of STAT3 by WB737 may increase the expression of NK cell activators and augment NK cell cytotoxicity against NKTL. In line with these studies, GSEA using the KEGG database also showed that WB737 significantly upregulated genes related to NK cell activation to activate the NK cell‐mediated cytotoxicity pathway. Consequently, these data suggest that WB737 may have the ability to strengthen NK cell cytotoxicity against NKTL and further functional studies are needed to verify WB737‐induced NK cell‐mediated antitumor function in the future.

In summary, WB737 is a novel STAT3 inhibitor that exhibits excellent anticancer effects against NKTL. The correlation between *STAT3*‐activating mutations and the sensitivity of NKTL to WB737 emphasizes the importance of precision medicine to identify patient subgroups that will benefit most from WB737 treatment. Given the high efficacy of WB737 for NKTL, there is an urgent need to translate these preclinical findings into the clinic to elucidate the potential clinical value of this target therapy.

## 4 MATERIALS AND METHODS

### 4.1 Ethics statement

All animal studies were conducted based on animal protocols approved by the Animal Care and Ethics Committee of Sun Yat‐Sen University and in accordance with the National of Health Guidelines on the Care and Use of Animals (SYSU‐IACUC‐2022‐000942).

### 4.2 Cell lines and reagents

Nine human NK/T‐cell lines, including NK‐S1, MEC04, YT, KAI‐3, KHYG1, NKYS, SNK6, SNK1, and NK92 cells, were used in our study. NK‐S1 cells were derived from an NK lymphoma xenograft.^51^ MEC04 was a gift from Dr. Paul Coppo and Dr. Philippe Gaulard, and YT was a gift from Dr. C. Clayberger. KAI‐3 and KHYG1 cells were purchased from JCRB Cell Bank, and NK92 cells were purchased from ATCC. SNK1, SNK6, and NKYS were kindly provided by Dr. Norio Shimizu. YT cells were cultured in IMDM (Life Technologies) with 20% fetal bovine serum (FBS, HyClone) and 1% sodium pyruvate (Life Technologies). NK‐S1 cells were grown in DMEM (Life Technologies) with 10% FBS and 10% horse serum (HS, Life Technologies). MEC04 cells were maintained in RPMI 1640 medium with 15% FBS and 50 IU/mL IL 2 (Miltenyi Biotec). All remaining cell lines were cultured in RPMI 1640 medium (Life Technologies) with 10% FBS and 100 IU/mL IL‐2. Ten percent HS was included in the NK92, SNK1, and SNK6 growth media. All culture media were supplemented with 1% penicillin‒streptomycin (P/S, Gibco BRL). All cultures were routinely assessed for mycoplasma contamination.

### 4.3 Molecular docking

The molecular docking study was performed by Schrodinger software to predict the molecular binding mode to the STAT3‐SH2 domain using the X‐ray crystal structure of the STAT3/DNA complex (PDB code: 1BG1). The protein preparation, generation of the receptor grid (box size: 10 Å × 10 Å × 10 Å), ligand preparation, and ligand docking were set with the default parameters. Eventually, the binding poses were generated by PyMOL software.

### 4.4 Microscale thermophoresis assay

The MST assay was performed as previously described.^52^ The Monolith NT.115 device (NanoTemper Technologies) was used to measure the binding affinities of WB737 with purified His‐STAT3‐SH2. The proteins were fluorescently labeled using the monolith His‐tag labeling kit (NanoTemper Technologies) based on the manufacturer's procedure and kept in an assay buffer comprising 50 mM HEPES, pH 7.4, 150 mM NaCl, and 0.1% (w/v) Nonidet P‐40 at a concentration of 200 nM. The labeled protein was mixed with the same volume of WB737 with 16 different serially diluted concentrations at room temperature. The samples were then loaded into premium capillaries (NanoTemper Technologies) and measured using the Monolith NT.115 device. Each assay was repeated two or three times. Data analyses were performed using MO Affinity Analysis v.2.2.4 software, and the figures were made by GraphPad Prism 8.0.

### 4.5 Cell viability assay

For each assay, 2000 cells were seeded on a 96‐well plate in triplicate and treated with the indicated concentrations of WB737 for 96 h. Cell viability was measured using the CellTiter‐Glo Luminescent Cell Viability Assay (Promega) following the manufacturer's instructions, and luminescence was read by the Infinite M200 plate reader (Tecan, Switzerland). IC_50_ values were calculated using GraphPad Prism 8.0.

### 4.6 Dual luciferase assay

The STAT3 response luciferase plasmid (vector: pGL4.47, Promega) and Renilla luciferase vector (phRL‐TK, Promega) were transiently transfected into 293T cells and NKYS cells using Lipofectamine 2000 (Invitrogen). After 24 h, the transfected cells were treated with the indicated concentrations of WB737 for 24 h with or without IL‐6. Renilla and firefly luciferase activities were detected using the Dual Luciferase Reporter Assay System (Promega), and the ratio of firefly to Renilla luciferase activity was calculated.

### 4.7 Plasmids and stable cell lines

STAT3/WT and STAT3/D661Y were constructed and integrated into the retroviral plasmid pMIGR1 (Addgene; plasmid no. 27490) as previously described.^17^ NK‐S1 cells were transduced with retroviral vectors harboring STAT3/WT and STAT3/D661Y to generate stable cell lines, and these cells were then sorted based on green fluorescent protein positivity using the BD Aria III Cell Sorter (BD Biosciences).

### 4.8 Cell cycle and apoptosis assays

A total of 2 × 10^5^ cells were seeded on a six‐well plate and treated with the indicated drugs for 72 h. The cells were then collected and washed with phosphate‐buffered saline (PBS). For the cell cycle assay, cells were fixed with 70% ethanol for more than 1 h. After washing with 1× PBS, 100 μg/mL RNase (Life Science) and 50 μg/mL propidium iodide (PI) (Sigma‒Aldrich) were added. For the apoptosis assay, the cells were stained with Annexin V and PI (BD Bioscience) following the manufacturer's instructions. The stained cells were analyzed by flow cytometry (SP6800, Sony) and quantified by FlowJo software (BD Bioscience).

### 4.9 Colony formation assay

A total of 2 × 10^4^ cells were cultured in growth media containing 0.4% methylcellulose (Sigma‒Aldrich), 10% FBS, and 1% P/S and layered on top of RPMI 1640 containing 0.6% agar, 10% FBS, and 1% P/S in a six‐well plate. After treatment with the indicated drugs for 10 days, the colonies were stained with iodonitrotetrazolium chloride (Sigma‒Aldrich) overnight. The experiment was performed in triplicate, and images were obtained from randomly selected areas using the Olympus microscope image system.

### 4.10 Western blot analysis

Cells (5 ×10^5^) were seeded on a six‐well plate, treated with the indicated drugs for 24 h and prepared in RIPA lysis buffer. Protein concentration was measured by Quick StartTM Bradford Protein Assay (Bio‐Rad), and 25 μg protein was loaded onsodium dodecyl sulfate polyacrylamide gel electrophoresis (SDS‐PAGE) and transferred to polyvinylidenefluoride (PVDF) membrane. The following primary antibodies were purchased from Cell Signaling Technology: pTyr705‐STAT3 (#9145s), pSer727‐STAT3 (#9134s), STAT3 (#9139s), Myc (CST, 5605), and GAPDH (CST, 2118). The secondary antibodies were  horseradish peroxidase (HRP)‐conjugated anti‐rabbit (GE Healthcare, #NA934‐1ML) and HRP‐conjugated anti‐mouse (GE Healthcare, #NA931‐1ML). The signals were detected using ChemiDoc MP System (Bio‐Rad).

### 4.11 Complex I activity assay

A Complex I enzyme activity assay kit (Abcam, ab109721) was used according to instructions from the manufacturer. Samples treated with or without WB737 were prepared using detergent solution and were diluted to a protein concentration of 500 μg/mL in incubation solution. These prepared samples were then loaded onto a 96‐well plate (200 μL/well) and incubated for 3 h at room temperature. The plate was washed three times with wash buffer, and then, 200 μL of assay solution was added into each well. Optical density (OD 450 nm) was measured in kinetic mode at room temperature for up to 30 min. The activity was expressed as the linear rate of increase in absorbance at OD 450 nm over time.

### 4.12 Mitochondrial oxygen consumption rate

The mitochondrial OCR was measured using an XFe/XF96 analyzer (Agilent Technologies) following the manufacturer's instructions. Cells (1.2 × 10^5^) were seeded on a Cell‐Tak (Corning, 354240)‐precoated XF 96 cell culture microplate (Seahorse Bioscience). Cells were then incubated in a CO_2_‐free environment for 1 h, and the indicated concentrations of WB737, 1.5 μM oligomycin, 0.5 μM trifluoromethoxy carbonylcyanide phenylhydrazone (FCCP), and 0.5 μM rotenone/antimycin A were sequentially injected into the microplate. OCR was measured under basal conditions and after injection of each test compound.

### 4.13 Immunohistochemical analysis

Mouse tumors were fixed in 10% formalin, embedded in paraffin, and sectioned into 5 μM slices. Antibodies specific to pTyr705‐STAT3 (CST, 9145), pSer727‐STAT3 (Abcam, ab32143), Ki67 (Zsbio Commerce Stor, ZA‐0502), and cleaved caspase 3 (CST, 9661s) were used. Images were captured using an Olympus microscope Imager. The staining area was scored using the following scale: 1, 0%−25% of tissue stained positive; 2, 26%−50% stained positive; 3, 51%−75% stained positive; and 4, 76%−100% stained positive. The staining intensity was scored using the following scale: 0, negative; 1, weak; 2, medium; and 3, strong. The IHC score was generated from three different areas of the slides, and the average score was calculated for each sample.

### 4.14 RNA sequencing and bioinformatics analysis

A total of 5 × 10^5^ cells were seeded on a six‐well plate, treated with or without 50 nM WB737 for 24 h and harvested for RNA extraction. Total RNA was extracted using the RNeasy Mini Kit (Qiagen) according to the manufacturer's protocol. Transcriptome sequencing libraries were prepared using TruSeq Stranded Total RNA Library Prep Gold (Illumina) following the manufacturer's instructions. Samples were then sequenced on the NovaSeq 6000 (lllumina) using the manufacturer's protocol. For data analysis, raw data were assessed for quality, and low‐quality reads were removed using fastp software (version 0.12.5).^53^ The clean reads were then aligned to the human reference genome (GRCh37, hg19) using STAR v2.7.0.^54^ Gene expression of the samples was analyzed by RSEM, a software for quantifying gene and isoform abundances from single‐end or paired‐end RNA‐Seq data, and differentially expressed genes were identified using edge R (*p*‐value < 0.05).^55^ R statistical programming was used for further analysis. The upregulated or downregulated genes upon WB737 treatment were subjected to KEGG pathway analysis and GSEA. Gene sets with a *p*‐value <0.05 and false discovery rate <0.25 were considered statistically significant.^56^

### 4.15 Real‐time RT‒qPCR

Cells were lysed using TRIzol Reagent (Thermo Fisher Scientific), and RNA was extracted using the RNeasy Mini Kit (Qiagen). Qantitative real time polymerase chain reaction （RT‒qPCR） was performed with 1 μg RNA using the TransScript All‐in‐One First‐Strand cDNA Synthesis SuperMix for qPCR kit (Transgen Biotech) and PerfectStart Green qPCR SuperMix kit (Transgen Biotech). Reactions were analyzed using a Real‐Time PCR Detection System (Bio‐Rad). The 18S rRNA level was used as an internal control to quantify the mRNA level. All experiments were performed in biological triplicates. The primer sequences used for qPCR are listed in Table S1.

### 4.16 In vivo studies

For the establishment of a YT‐derived xenograft model, 1.5 × 10^7^ YT cells were suspended in a mixture containing 75 μL of PBS and 25 μL of Matrigel (Corning, 354234) and then inoculated subcutaneously into nude mice. Tumor volume and body weight were measured every other day, and tumor volume was calculated as (width)^2^ × length/2. WB737 was dissolved in sterile water with 0.5% methyl cellulose (MC). When tumors reached 200−300 mm^3^ (small tumors), they were first randomly divided into control and drug treatment groups: treated with 0.5% MC in sterile water (*n* = 5 p.o.), WB737 (*n* = 5, 5 mg/kg/day, p.o.). When tumors in the control group reached 1000 mm^3^ (large tumors), they were randomized into another control and drug treatment groups: treated with 0.5% MC (*n* = 5, p.o.), WB737 (*n* = 5, 5 mg/kg/day, p.o.). Tumors were monitored for approximately 4 weeks. Once the tumor volume reached approximately 2000 mm^3^, the tumors were excised for further analysis. Female BALB/c nude mice (5−6 weeks old) were purchased from Beijing Vital River Laboratory Animal Technology Company.

### 4.17 Statistical analysis

At least three biological replicates were performed for all experiments, and data are presented as the mean ± standard deviation (SD)/standard error of mean (SEM). All statistical analyses were performed using GraphPad Prism version 8.0. An unpaired Student's *t*‐test was used to analyze the significant difference between two groups, while a two‐way analysis of variance was used to determine the significant difference in dose‒response curves, as indicated in the figure legends. The data are reported as the means ± SDs of three independent experiments. In all statistical tests, statistical significance was considered *p* < 0.05 unless stated otherwise. For all figures: ^*^ *p* < 0.05; ^**^ *p* < 0.01; ^***^ *p* < 0.001.

## AUTHOR CONTRIBUTIONS

J.T. and Y.C. designed and conceived the study. W.Z. discovered and synthetized WB737. Y.W. conducted most of the experiments and prepared the manuscript. J.T. and Y.C. supervised the project and revised the manuscript. Y.S., L.L., H.C., and Z.Y. contributed to the technical support and animal work. J.C. and P.D. performed the RNA sequencing data analysis. J.C. and P.W. provided material support. J.T., J.H.H., B.T.T., Z.Y., S.T.L., M.L., Y.C., W.L., and C.K.O. provided a critical reading of the manuscript. All the authors have given their consent to publish this study.

## CONFLICT OF INTEREST STATEMENT

The authors declare that there is no conflict of interest that could be perceived as prejudicing the impartiality of the research reported. Authors Mingyao Liu, Wenbo Zhou, and Huang Chen are employees from Shanghai Yuyao Biotech Co., Ltd., but have no financial and non‐financial interests to disclose.

## ETHICS STATEMENT

Human subjects were not used in this study. All preclinical investigations involving animals were carried out in line with ethical standards and according to the approved protocol by IACUC (approval number SYSU‐IACUC‐2022‐000942).

## Supporting information

Supporting information

## Data Availability

The dataset generated during the current study is available from the corresponding author on reasonable request. RNA sequencing data have been deposited in the GEO database under project accession number GSE202944.
