# Supplementary material for: Preclinical characterization of WB737, a potent and selective STAT3 inhibitor, in natural killer/T‐cell lymphoma
Source: MedComm (2020). 2023 Jun 16;4(4):e284. doi: 10.1002/mco2.284 (PMC10274570; doi:10.1002/mco2.284)
Supplement: Supplementary file 1 — Supporting information [file MCO2-4-e284-s001.pdf]

Preclinical characterization of WB737, a potent and selective STAT3 inhibitor, in natural killer/T-cell lymphoma

Yali Wang,[1,*] Wenbo Zhou,[2,3,*] Jianfeng Chen,[1] Jinghong Chen,[4] Peng Deng,[1] Huang Chen,[2,3] Yichen Sun,[5] Zhaoliang Yu,[6] Diwen Pang,[7] Lizhen Liu,[7] Peili Wang,[1] Jing Han Hong,[8] Bin Tean Teh,[8,9] Huiqiang Huang,[1] Wenyu Li,[7] Zhengfang Yi,[2] Soon Thye Lim,[10] Yihua Chen,[2] Choon Kiat Ong,[8,11] Mingyao Liu,[2,3,#] Jing Tan[1,9,#]

Affiliations

1.State Key Laboratory of Oncology in South China, Collaborative Innovation Center of Cancer Medicine, Sun Yat-sen University Cancer Center, Guangzhou, China

2.Shanghai Key Laboratory of Regulatory Biology, Institute of Biomedical Sciences and School of Life Sciences, East China Normal University, Shanghai, 200241, China

3.Shanghai Yuyao Biotech Co., Ltd., Shanghai, 200241, China

4.Department of Medical Oncology, The Affiliated Cancer Hospital of Zhengzhou University & Henan Cancer Hospital, Zhengzhou, China

5.Department of Laboratory Medicine, Guangzhou First People's Hospital, School of Medicine, South China University of Technology

6.Department of Colorectal Surgery, the Sixth Affiliated Hospital, Sun Yat-sen University, 510655, Guangzhou, Guangdong, P. R. China

7.Guangdong Provincial People's Hospital, Guangdong Academy of Medical Sciences, School of Medicine, South China University of Technology Guangzhou, China

8.Cancer and Stem Cell Biology Program, Duke-NUS Medical School, Singapore

9.Laboratory of Cancer Epigenome, Division of Medical Sciences, National Cancer Centre Singapore, Singapore

10.Director's office, National Cancer Centre Singapore, Singapore

11.Division of Cellular and Molecular Research, National Cancer Centre Singapore, Singapore

*These authors contributed equally to this study.

Dr. Jing Tan
State Key Laboratory of Oncology in South China, Collaborative Innovation Center of Cancer Medicine, Sun Yat-sen University Cancer Center, 651 East Dongfeng Road, Guangzhou 510060, PR China; E-mail: tanjing@sysucc.org.cn;

Dr. Mingyao Liu
Shanghai Key Laboratory of Regulatory Biology, Institute of Biomedical Sciences and School of Life Sciences, East China Normal University, Shanghai, 200241, China. E-mail: myliu@bio.ecnu.edu.cn;

Fig.S1

A

Acquisition Parameter

Source Type	ESI	Ion Polarity	Positive	Set Nebulizer	1.5 Bar
Focus	Active	Set Capillary	4500 V	Set Dry Heater	180 °C
Scan Begin	50 m/z	Set End Plate Offset	-500 V	Set Dry Gas	6.0 l/min
Scan End	1350 m/z	Set Collision Cell RF	700.0 Vpp	Set Divert Valve	Waste

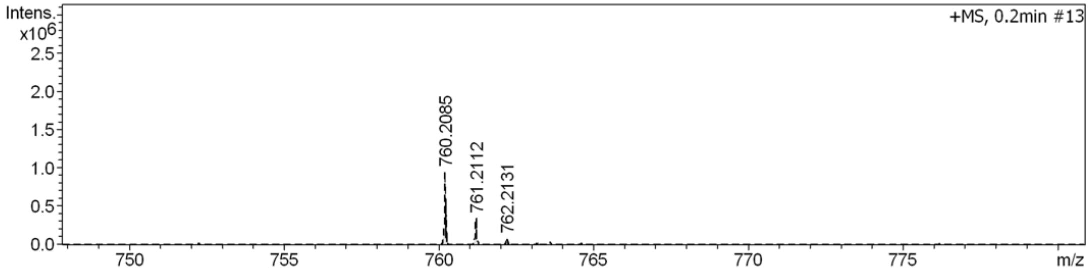

B **C**

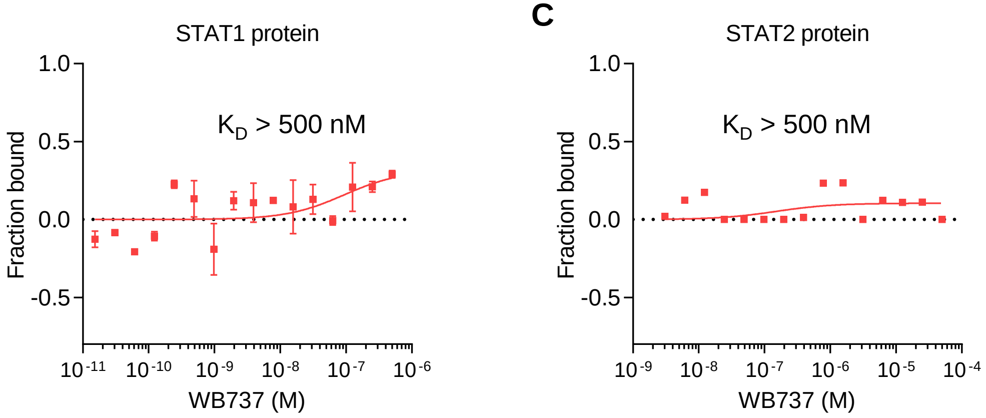

Figure S1. Identification of WB737. (A) The high-resolution mass spectrum (HRMS) of WB737. HRMS (ESI): calculated for C36H29F6N7NaO4, m/z = 760.2077, [M + Na]+, found 760.2085. High-resolution mass spectrum (HRMS) was gathered on a Bruker MicroTOF-Q III LC MS instrument operating in electrospray ionization (ESI). **(B and C)** MST assays. WB737 was serially diluted and mixed with equal volumes of labelled His-STAT1 or STAT2 protein. The MST signal was measured, and the data were analysed using MO analysis software.

Fig.S2

A

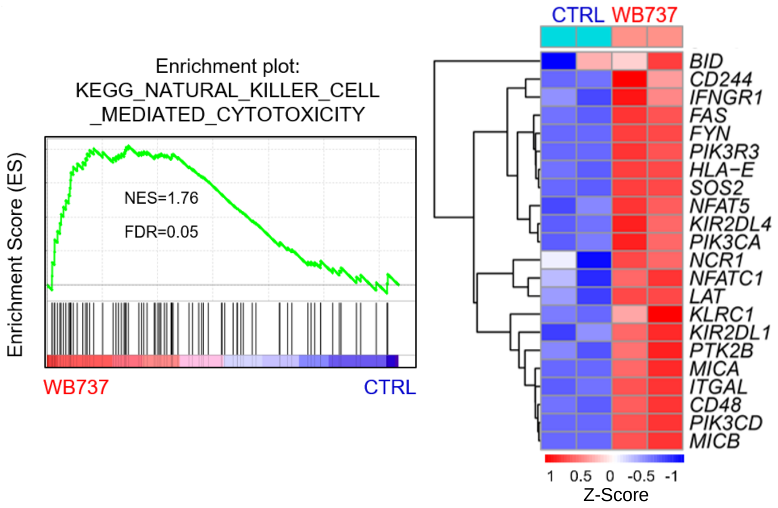

Figure S2. WB737 upregulated the NK cell-mediated cytotoxicity pathway in NKYS cells. (A) GSEA using the KEGG pathway database revealed that the NK cell-mediated cytotoxicity pathway was significantly enriched in the upregulated genes (left panel). The heatmap indicates the top 20 upregulated genes of the NK cell-mediated cytotoxicity pathway in NKYS cells treated with WB737 (right panel). NES denotes the normalized enrichment score; FDR denotes the false discovery fate. Z-Score denotes the deviation from the mean by standard deviation units. Red colour indicates up-regulated expression, whereas blue colour indicates down-regulated expression.

Fig.S3

A

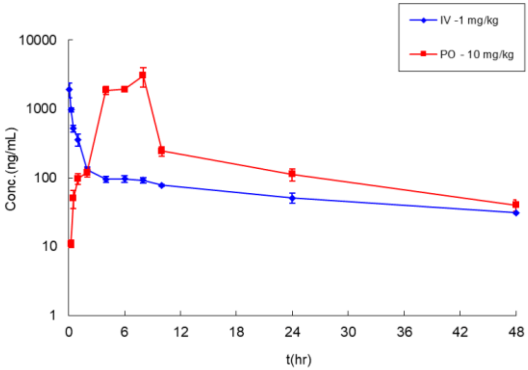

Dosing	$t_{1/2z}$	T_{max}	C_{max}	$AUC_{(0-t)}$	$AUC_{(0-\infty)}$	V_z	CL_z	$MRT_{(0-t)}$	F
	h	h	ng/mL	ng/mL*h	ng/mL*h	mg/Kg	mL/hr/kg	h	%
IV	29.08	0.08	1919.39	3734.27	5032.29	8336.75	198.72	13.76	/
PO	14.70	8.00	3040.92	18509.24	19355.74	/	/	9.77	38.46

B

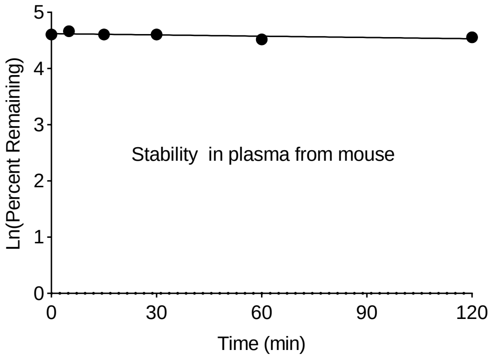

C

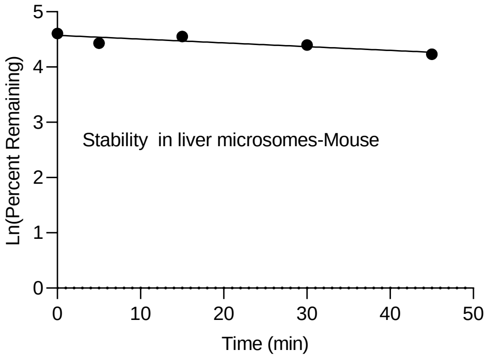

Figure S3. The pharmacokinetic properties of WB737. (A) Pharmacokinetic data of WB737 in mice (n=3) after oral (10 mg/kg) and intravenous (1 mg/kg) administration. The half-life of WB737 in both the plasma **(B)** and liver microsomes. **(C)** from mice was 911.03 minutes. **(A)** $T_{1/2}$ denotes elimination half-life, T_{max} denotes time of C_{max}, C_{max} denotes maximu observed concentration, occurring at T_{max}, AUC_{0-t} denotes area under the curve from the time of dosing to the last measurable concentration, $AUC_{0-\infty}$ denotes AUC from dosing time extrapolated to infinity, based the last predicted concentration, V_z denotes apparent volume of distribution, CL_z denotes apparent clearance, MRT_{0-t} denotes the average time a molecule stays in the body and F denotes bioavailability from the time of dosing to the last measurable concentration

Table S1

Gene	Forward primer sequence	Reverse primer sequence
c-Myc	CCTGGTGCTCCATGAGGAGAC	CAGACTCTGACCTTTTGCCAGG
18S	AACCCGTTGAACCCCATT	CCATCCAATCGGTAGTAGCG
MTCO3	ACGGCATCTACGGCTCAACA	TGGCGGATGAAGCAGATAGTGA
MTND1	CCACCTCTAGCCTAGCCGTTTA	GGGTCATGATGGCAGGAGTAAT
MTATP6	TAGCCATACACAACACTAAAGGACGA	GGGCATTTTTAATCTTAGAGCGAAA
MTCYB	ATCACTCGAGACGTAAATTATGGCT	TGAACTAGGTCTGTCCCAATGTATG
MTND2	GCCCTAGAAATAAACATGCTA	GGGCTATTCCTAGTTTTATT

Table S1. The primer sequences used for RT-qPCR.
